# Chinese Consumers’ Herd Consumption Behavior Related to Korean Luxury Cosmetics: The Mediating Role of Fear of Missing Out

**DOI:** 10.3389/fpsyg.2020.00121

**Published:** 2020-02-19

**Authors:** Inwon Kang, Xue He, Matthew Minsuk Shin

**Affiliations:** ^1^Department of International Business and Trade, Kyung Hee University, Seoul, South Korea; ^2^Department of International Trade, College of Social Science, Konkuk University, Seoul, South Korea

**Keywords:** Chinese consumer, Korean luxury cosmetics brand, herd consumption behavior, fear of missing out, emotional needs

## Abstract

Chinese consumers’ lavish and collective spending on Korean luxury cosmetics brands is well documented. This study examines why this consumption behavior occurs, hypothesizing that it is driven by a “fear of missing out” (FoMO). In other words, in order to derive psychological comfort, consumers with high FoMO may be prone to developing high brand involvement, leading to their collective consumption of certain luxury brands. In consumer studies, such collective consumption behavior is referred to as herd behavior. Thus, the main research question of this study is, “why do Chinese consumers show herd consumption behavior toward certain luxury brands?” We propose that consumers who are attracted to luxury brands and possess high FoMO will develop higher brand involvement, leading to herd consumption behavior toward such brands. To validate this proposition, this study surveyed Chinese consumers. The collected data were analyzed using the structural equation modeling (SEM) method, conducted within SPSS and AMOS. The results provide theoretical explanations as to why Chinese consumers indulge in collective obsession-like stockpiling consumption toward certain luxury brands, as well as several managerial implications related to this behavior.

## Introduction

Chinese consumers are known to spend significant amounts on South Korean luxury cosmetic brands, or so-called K-Beauty brands (Korea Economy, 2019). This spending on K-beauty items goes beyond simply lavish shopping, often reaching a level of stockpiling. For example, it is not unheard of for Chinese customers to simply empty the shelves of duty-free shops in Jeju-do, a tropical island in South Korea (The Korea Herald, 2019). Of course, this occurs in other areas as well. In the past, Chinese consumers have shown similar stockpiling behavior toward European luxury brands, Japanese electronics, and other trendy brands of the time (McKinsey Company, 2019). This study aims to understand the reasons for Chinese consumers’ collective stockpiling consumption behavior related to certain luxury brands.

Consumers often form deep attachments with a brand. This is known as brand involvement, and is defined as a consumer’s perceived attachment to a brand based on his or her interests, values, and needs (Brakus et al., 2009). Chinese consumers demonstrate particularly high brand involvement with luxury brands (McKinsey Company, 2019). A luxury brand represents unique attributes, such as symbolism, hedonism, quality, utility, and other exclusive values (Kang et al., 2014). As such, consumers who show high involvement with such goods often do so to demonstrate success, be set apart, embellish, and/or make a statement (Kastanakis and Balabanis, 2012). However, these characteristics, which have been examined in general, do not fully explain Chinese consumers’ collective stockpiling consumption. Therefore, there must be characteristics particular to Chinese consumers that lead to such behavior.

Chinese consumers scored 20 on the Hofstede individualism scale. This score is among the lowest individualism in the world, and indicates that the Chinese are extreme collectivists. A recent characteristic of the consumption pattern in China is that the largest portion of Chinese domestic consumption is led by the “neo-middle class,” who are highly educated, have stable professions, and earn an annual salary of USD 20,000–80,000 (Maeil Business Newspaper, 2019). This group is known to portray themselves as the neo-middle class through their consumption or ownership of foreign luxury brands (Maeil Business Newspaper, 2019). Another new consumption culture in China is called “loneliness consumption.” According to the KOTRA Report (2019), 14% of households in China are single-person households. Loneliness consumption is intended to alleviate feelings of loneliness, as well as the stress of living away from family members, alone in a metropolitan city (South China Morning Post, 2018). An important feature of loneliness consumption is that it follows trends, as those making such purchases attempt to derive psychological comfort by being part of the group represented by the current fashion trends (People’s Daily Online, 2019). In other words, the major consumption pattern in China can be identified as being based on the desire to be part of the neo-middle class, as well as by the attempts of consumers to alleviate their loneliness by following consumption trends. In the consumer behavior literature, such a consumption culture can be understood in terms of the “fear of missing out” (FoMO). FoMO refers to a persistent fear that others may have positive experiences while one is absent (Kang et al., 2019). Thus, we assume that FoMO might be a characteristic that explains Chinese consumers’ collective stockpiling consumption.

As noted earlier, the general characteristics of consumers who show high involvement in luxury brands include their desire to fulfill their psychological needs through luxury purchases (Voss et al., 2003; Naylor et al., 2008; Kastanakis and Balabanis, 2012). Among Chinese consumers who exhibit these characteristics, those with high FoMO might show obsession-like high brand involvement toward certain luxury brands, and thus stockpile these brands. In consumer studies, such collective consumption behavior is referred to as herd behavior. Consumer herd behavior is defined as purchase behavior that follows that of relevant others (e.g., Watts and Dodds, 2007; Spiller and Belogolova, 2016; Kang et al., 2019). Given our research question of why Chinese consumers show herd consumption behavior toward certain luxury brands, we propose that consumers who are attracted to luxury brands and exhibit high FoMO develop higher involvement with such brands, leading to herd consumption behavior.

To validate this proposition, we surveyed Chinese consumers. The survey questionnaire asked respondents to rate their general psychological needs and levels of FoMO. Then, the respondents were asked to evaluate their involvement with preselected Korean luxury cosmetics brands, as well as the likelihood of their herd consumption toward these brands. The collected data were analyzed using the structural equation modeling (SEM) method, conducted through SPSS and AMOS. Our results provide theoretical explanations as to why Chinese consumers indulge in such collective obsession-like stockpiling consumption with certain luxury brands, as well as managerial implications related to this behavior.

The remainder of this paper is organized as follows: section “Literature Review” reviews the related literature, section “Materials and Methods” describes our research method, section “Results” presents our results, and section “Conclusion and Discussion” addresses our research question based on our results and provides theoretical and managerial implications.

## Literature Review

### Consumer Herd Behavior

On an individual level, herd behavior refers to the phenomenon of an individual following others’ behaviors (e.g., Gao et al., 2018; Liu and Yang, 2018; Shantha, 2019). In psychology, herd behavior is understood as a behavior that irrationally and emotionally follows that of a crowd; some examples are riots, religious gatherings, and sporting events (e.g., Berger and Heath, 2008; Puaschunder, 2018; Allie and Ajiboye, 2019). In the field of consumer behavior, herd behavior is defined as a change in a consumer’s product evaluation, purchase intention, or purchase behavior as a result of exposure to similar behaviors of relevant others (e.g., Watts and Dodds, 2007; Spiller and Belogolova, 2016; Kang et al., 2019).

Existing studies report that consumers, although they make rational choices, engage in herd behavior because they are cognitively lazy (e.g., Spiller and Belogolova, 2016; Liu and Yang, 2018; Allie and Ajiboye, 2019). As such, they often prefer simple to complex solutions; for example, they may prefer to rely on heuristics, such as group consensus, when making a decision (e.g., Puaschunder, 2018; Salcedo and Jiménez-Leal, 2019; Shantha, 2019). Numerous studies have shown that individuals employ such heuristics over a rational inspection of details, but then irrationally imitate group behaviors (e.g., Watts and Dodds, 2007; Berger and Heath, 2008; Kang et al., 2019). Table 1 summarizes the relevant previous research on herd behavior.

**TABLE 1:** Prior research on herd behavior.

Research	Antecedents	Field of study
Allie and Ajiboye, 2019	Adaptation	Organizational psychology
Berger and Heath, 2008	Identity signal; group similarity	Social psychology
Gao et al., 2018	Peer pressure	Psychology
Kang et al., 2019	Fear of missing out	Consumer behavior
Liu and Yang, 2018	Physical/psychological ease	Consumer behavior
Puaschunder, 2018	Desire to minimize losses and maximize profits	Organizational psychology
Salcedo and Jiménez-Leal, 2019	Desire to reduce risk in decision making	Psychology
Shantha, 2019	Peer learning experience	Consumer behavior
Spiller and Belogolova, 2016	Quality assurance	Consumer behavior
Watts and Dodds, 2007	Influences: networks; public opinion	Consumer behavior

Despite the significant contributions of previous studies on illustrating herd behavior, few have examined Chinese consumers’ herd consumption of Korean luxury cosmetic brands, or attempted to explain this behavior from the perspective of Chinese consumers’ psychological motivations for consuming such brands.

### Luxury Categories and Values

Existing studies suggest that luxury brands fall into three categories: democratic, elitist, and charismatic. Each of these categories includes various luxury values (e.g., Bian and Forsythe, 2011). The democratic category incorporates the necessity aspect of luxury brands, which conveys physical value based on practicality, quality, and uniqueness (Wetlaufer, 2001; Tynan et al., 2010; Li et al., 2011). The elitist category is an additional condition that conveys emotional value from materialism, sentimentalism, and hedonism (Prendergast and Wong, 2003; Tynan et al., 2010; Laroche et al., 2011). The charismatic category offers social value through status, authority, and self-expression (Wilcox et al., 2009; Laroche et al., 2011; Miller and Mills, 2011). The categories representing emotional and social value represent that which is unique to luxury brands. The following section discusses how such values fulfill consumers’ psychological needs.

### Satisfying Psychological Needs Using Luxury Brands

#### Emotional Value of Luxury Brands

Emotional needs form a core component of consumer satisfaction (Andreu et al., 2015), which means the satisfaction construct cannot be fully understood or explained without accounting for effect in the form of consumer emotion (Saeidi et al., 2015). Thus, it is necessary to consider consumers’ emotional satisfaction when examining their consumption behavior related to luxury brands. In prior work on luxury brand consumption, four dimensions have been proposed as critical to creating value: symbolic/expressive, experiential/hedonic, utilitarian/functional, and cost/sacrifice (Kang et al., 2014). In this study, we focus on luxury brand value derived from hedonic value and symbolic value.

Hedonism is typically regarded as a form of egoism, where pleasure and the avoidance of pain are the dominant motives for action (Kang et al., 2014). According to Kang et al. (2014), hedonism can explain all motivations for buying. A hedonic product is purchased primarily for sensory satisfaction and emotional purposes (i.e., entertainment and enjoyment). Such hedonic products attempt to arouse emotions such as taste, symbolism, and sensory experience (Juaneda-Ayensa et al., 2016). Juaneda-Ayensa et al. (2016) suggest that in contemporary society, consumers have the right to seek an enjoyment experience. Hedonistic consumption may arise from the purchase experience, the consumption of the goods or services purchased (Kang et al., 2014), and the possession of certain goods (Juaneda-Ayensa et al., 2016). For example, such products can bring their owners the opportunity to showcase their style and taste. They also enable potential customers to imagine the enjoyable experience of shopping for the items, although such thoughts tend to be unrealistic.

Smith and Colgate (2007, p. 10) define consumers’ symbolic needs as the “extent to which customers attach or associate psychological meaning to a product.” Several prior studies note that luxury goods appeal to consumers’ self-concept and self-worth (e.g., Naylor et al., 2008; Kang et al., 2014). The symbolic interaction theory is based on the social nature of self-definition (Carbonero et al., 2017). It is defined largely through individuals’ attitudes toward themselves and is determined by how others evaluate them. Status related to symbolism that is important to an individual is more likely to influence his or her actions and guide appropriate behavior. The findings of an empirical study conducted by Kang et al. (2017) emphasize that, similar to functional or high-value brands and products, functionality and symbolism influence consumers’ evaluations of products. The expressiveness of such products is similar to the symbolic practical value, whereas the utilitarian value of the aforementioned brands lies approximately halfway along the symbolic scale.

#### Social Value of Luxury Brands

Social identity is reported to be a strong predictor of human behavior, including the innovative behavior of consumers (Kastanakis and Balabanis, 2012). Social identity refers to a perception of oneness with or belongingness to a group, and people who belong to such groups describe themselves in terms of the characteristics of the group (Pan et al., 2019). Many studies have examined how consumers’ feelings of belonging to a group influence their herd behavior. Such feelings include self-esteem, self-confidence, a need for uniqueness, adaptability, innovativeness, and a need to be correct. Of these, consumers’ self-esteem needs and recognition needs are considered significant in terms of their herding behavior related to luxury brands (Kastanakis and Balabanis, 2012).

Self-esteem assesses an individual’s perception of himself or herself (Fuller et al., 2006). In psychology, numerous studies have examined self-esteem and its connections with a person’s cognition, emotions, and behavior (Wang et al., 2017). According to Fuller et al. (2006), self-esteem refers to adolescents’ evaluations of their own worth or their satisfaction with themselves, and is divided into three dimensions of the self: physical appearance, romantic attractiveness, and the ability to develop and keep close friendships. Wang et al. (2017) proposes that self-esteem refers to a comparatively steady mental state that reflects people’s social reality and influences their cognition and behavior. People with high self-esteem tend to be more resilient to external influences than are those with low self-esteem. A person with high self-esteem considers their true self to be positive, seeks to confirm their true appearance through their preferences and values, and accepts their own flaws (Fuller et al., 2006). In other words, self-esteem has been viewed as a one-dimensional construct that represents an individual’s overall positive or negative attitude toward themselves.

Recognition needs refer to the psychological state in which a consumer perceives, feels, or evaluates their sense of belonging to a brand (Gordon and Luo, 2011). Consumers can make an inference about the quality and value of a brand using information about its external evaluation provided by the brand (Shin et al., 2018). Brands may also directly reflect their differences from competitors through their internal behavior. The identification of luxury goods is a brand awareness of luxury brands (Kang et al., 2014). The purchase and consumption of such brands enable consumers to build an identity for themselves. If consumers believe that a brand reflects their identity, they combine the features of the brand with self-awareness and self-definition (Gordon and Luo, 2011). Therefore, when consumers identify with a brand, they connect with it, allowing for an enjoyable shopping experience and playing a crucial role in the selection of the product (Palací et al., 2019). This intrinsic connection with the brand encourages consumers to participate in a higher level of information sharing (i.e., word-of-mouth marketing), thus achieving the brand’s interests (Shin et al., 2018). Therefore, brand awareness can be combined with other structures to attract additional consumers (e.g., by expressing love for the brand). Furthermore, the evaluation of brand recognition and brand attitudes is very similar, which may be caused by the results of the recognition.

### The Role of FoMO

Fear of missing out describes people’s fear of detachment and the desire to stay continually connected with what others are doing (Kang et al., 2019). It is usually regarded as a personality trait that leads to self-initiated behavior, and may cause individuals to neglect activities that are more compatible with their interests, goals, and values (Eide et al., 2018). In consumer behavior, FoMO is viewed as a social phenomenon that leaves people feeling alienated from their current experiences (Kang et al., 2019). Those who are isolated from society may experience anxiety as a result, increasing their tendency to imitate and follow the behavior of others (Spiller and Belogolova, 2016), such as choosing a product or brand (Kang et al., 2019). In other words, FoMO plays an important role in the consumption process in terms of driving purchases (Hodkinson, 2019). Additionally, many studies have found that Internet dependency is an indicator of high FoMO (Al-Menayes, 2015). A global trend survey (Ipsos Reid, 2017) and worldwide internet usage surveys (Statista, 2019a) report that the Chinese show a high dependency on the Internet and social media, indicating a high level of FoMO (see Table 2). Therefore, we propose that Chinese consumers’ high levels of FoMO influence their level of involvement with a brand. Brand involvement should also be considered as the degree of interest of a consumer in a product category on an ongoing basis. This is commonly defined as a consumer’s enduring perceptions of the importance of a product category, based on their inherent needs, values, and interests (Brakus et al., 2009).

**TABLE 2:** Internet use and dependency, by country.

	Percentage of respondents who cannot imagine life without the Internet	Number of Internet users, as of March 2019 (in millions)	Number of Internet users with high dependency (estimate)
China	77	829	638.33
India	82	560	459.2
United States	73	293	213.89
Japan	62	118	73.16
Russia	66	110	72.6
Germany	73	79	57.67
United Kingdom	78	63	49.14
France	64	60	38.4
Italy	62	55	34.1

### Chinese Consumers’ Involvement With Korean Luxury Cosmetic Brands

Brand involvement refers to a consumer’s perceived attachment to a brand based on their interests, values, and needs (Brakus et al., 2009). Chinese consumers’ involvement with Korean luxury cosmetic brands significantly affect their herd consumption of these brands. Depending on their level of involvement, Chinese consumers differ greatly in terms of the extensiveness of their purchase-decision process, as shown in the number of attributes they use to compare Korean luxury cosmetic brands, the length of their choice processes, and their willingness to reach a maximum or a threshold level of satisfaction. In addition, empirical studies on the relationship between individuals and groups have demonstrated that individual psychological processes are always affected by social influences. Social influences can be one of two categories: informational and normative (Kuan et al., 2014). Informational influences are considered indicators of reality and are based on information received from others. Normative influences are those that cause individuals to conform to the expectations of others (Chen, 2008). In consumer behavior, consumers often follow other people’s purchase decisions after asking for and gathering information. However, others may purchase a certain brand after being influenced by the need for another’s approval and acceptance (Liu and Yang, 2018). Thus, we examine Chinese consumers’ herd consumption behavior from these two aspects. For different involvement levels with Korean luxury cosmetic brands, informational consumers conform to others’ purchase beliefs and decisions because of their knowledge and expertise. In contrast, normative consumers follow the perceived expectations of other consumers in order to fit in with the social group.

### Hypotheses

#### Emotional Needs and Involvement With Korean Luxury Cosmetic Brands

Hedonic consumption relates to the multisensory, fantasy, and emotive aspects of one’s experience with products (Kang et al., 2014). It involves a response related to emotional arousal, which includes feelings such as delightfulness, excitement, and thrill (Voss et al., 2003). Chinese consumers who seek such aspects desire personal enjoyment by consuming Korean luxury cosmetic goods.

The symbolic meaning that a brand possesses can help consumers achieve their fundamental identity goals (Gordon and Luo, 2011). Furthermore, consumers usually choose a brand as a symbol of their preferences related to society and their social interrelations with others. Naylor et al. (2008) argue that brands are symbolic resources used by consumers to communicate the self to others. Chinese consumers with higher symbolic needs may try to express their desirable lifestyle within their social setting by purchasing Korean luxury cosmetic products, thus increasing their endorsement of and interest in such brands. Therefore, we propose the following hypotheses:

H1: Chinese consumers’ hedonic needs and their involvement with Korean luxury cosmetic brands have a positive relationship.
H2: Chinese consumers’ symbolic needs and their involvement with Korean luxury cosmetic brands have a positive relationship.

#### Social Identity Needs and Involvement With Korean Luxury Cosmetic Brands

Consumers form their ideal identity and self-image by purchasing a specific brand, among other things (Pan et al., 2019). The closer consumers approach their ideal self, the better they feel, which helps raise their self-esteem (Fuller et al., 2006). Individuals usually actively decide on a brand purchase and brand evaluation (Gordon and Luo, 2011). A need for recognition may lead to a high involvement with a particular brand (Kang et al., 2014). In other words, a brand is perceived as attractive when it helps a consumer express themselves and identify with the brand. Thus, Chinese consumers with higher self-esteem and recognition needs may try to express their desirable lifestyle within their social setting by purchasing Korean luxury cosmetic products, thus increasing their endorsement of and interest in such brands. Therefore, we propose the following hypotheses:

H3: Chinese consumers’ self-esteem needs and their involvement with Korean luxury cosmetic brands have a positive relationship.
H4: Chinese consumers’ recognition needs and their involvement with Korean luxury cosmetic brands have a positive relationship.

#### Korean Luxury Cosmetic Brand Involvement and Herd Behavior

Normative herd behavior, the purpose of which is to be part of a group, is influenced mainly by pre-existing characteristics of the consumer (Chen, 2008; Kuan et al., 2014). The informational type, on the other hand, is related mainly to qualities inherent in the virtual community (Chen, 2008; Liu and Yang, 2018). Consumers’ brand involvement has been used extensively as an explanatory variable when examining influences on consumer behavior (Brakus et al., 2009). Studies have found that the level of involvement determines the depth, complexity, and extensiveness of cognitive and behavioral processes during the consumer choice process (Chen, 2008; Kuan et al., 2014). Thus, Chinese consumers’ brand involvement may be a significant factor in their decisions to purchase certain Korean luxury cosmetic brands. Accordingly, the following hypotheses are proposed:

H5: Chinese consumers’ involvement with Korean luxury cosmetic brands and their informational herd behavior have a positive relationship.
H6: Chinese consumers’ involvement with Korean luxury cosmetic brands and their normative herd behavior have a positive relationship.

#### The Mediating Role of FoMO Between Psychological Needs and Brand Involvement

Studies have found that people may change what they typically do or purchase because of social pressures and a fear of being excluded (e.g., Kang et al., 2019). As members of society, consumers have connections with family, schoolmates, colleagues, neighbors, and social groups in their daily lives. Individuals with a high level of FoMO tend to be more aware of what others are doing, and seek to meet their needs in order to connect with others (Eide et al., 2018). The expectation of praise from others may reflect an individual’s need to seek novelty. The hedonic value of a brand increases consumers’ attention to the brand, and FoMO implies their involvement with the brand is high. Thus, Chinese consumers’ hedonic and symbolic needs related to Korean luxury cosmetics significantly influence their endorsement of and interest in certain brands.

Furthermore, the needs of self-esteem and recognition are considered more applicable to consumers with a high level of FoMO (Hodkinson, 2019). FoMO causes a strong need for interpersonal attachments as a fundamental human motivation, for example, the need for others to identify their status, and the need for recognition from others (Kang et al., 2014). Thus, Chinese consumers’ need for self-esteem and recognition affect their involvement in specific Korean luxury cosmetic brands if they feel strong FoMO. As such, we propose the following hypotheses:

H7a: The relationship between Chinese consumers’ hedonic needs and their involvement with Korean luxury cosmetic brands is stronger in the high-FoMO group than it is in the low-FoMO group.
H7b: The relationship between Chinese consumers’ symbolic needs and their involvement with Korean luxury cosmetic brands is stronger in the high-FoMO group than it is in the low-FoMO group.
H8a: The relationship between Chinese consumers’ self-esteem needs and their involvement with Korean luxury cosmetic brands is stronger in the high-FoMO group than it is in the low-FoMO group.
H8b: The relationship between Chinese consumers’ recognition needs and their involvement with Korean luxury cosmetic brands is stronger in the high-FoMO group than it is in the low-FoMO group.

### Research Model

**FIGURE 1:**
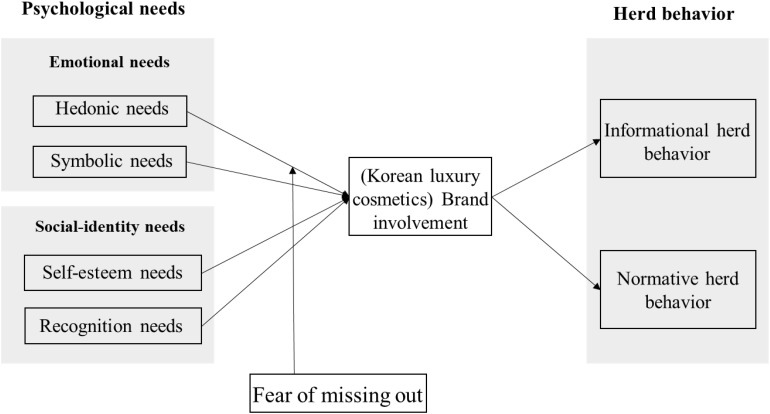
Research model.

Figure 1 shows the research model for the eight constructs and their theoretical relationships, as discussed above.

## Materials and Methods

### Sampling

We selected Chinese respondents who are familiar with Korean luxury cosmetics and who frequently purchase such goods. The five major Chinese cities of Beijing, Shanghai, Tianjin, Dalian, and Shenyang were examined. Quota sampling was used to collect different samples based on gender, age, and occupation. Although quota sampling is a non-probability sampling method, the samples accurately reflect the characteristics of the population when units are selected correctly. Here, we wish to examine Chinese consumers’ collective stockpiling purchasing behavior as a form of herd consumption. Therefore, we selected two top-selling Korean luxury brands (i.e., K-Beauty) that Chinese consumers are known to purchase in significant quantities, Whoo and Sulwhasoo (Korea Economy, 2019), as the basis for the survey. We had respondents evaluate their perceptions of themselves and of the two brands. These brands are the most popular in China because of the perceived significance of their symbolism and identification.

Because the respondents are Chinese consumers, the survey instruments were translated from English into Chinese, and a pre-test was conducted on 30 Chinese students in Korea. Accordingly, any problems or difficult expressions were resolved. The questionnaires were distributed through an online survey in China. In total, 293 questionnaires were returned, 279 of which were valid and used in our analysis; the remaining 14 were not answered correctly.

### Constructs and Measurement Items

For the measurements used, operational definitions were specified on each construct to meet the purpose of the study, and then the measurement items were modified based on previous studies. Questions were constructed on a five-point Likert scale, with answers ranging from “strongly disagree” (=1) to “strongly agree” (=5). In total, the questionnaire comprised 25 items for the eight constructs and three demographic questions. The measurement items were collected from various studies and modified to match the current research context. The constructs and measurement items are shown in Table 3.

**TABLE 3:** Measurement items.

Constructs	Measurement items	Source
Hedonic needs	I seek delightful experiences	Voss et al., 2003
	I seek exciting experiences	
	I seek thrilling experiences	
Symbolic needs	I like to have my friends envy me	Naylor et al., 2008
	I like to have experiences that I want to tell my friends about	
	I like to feel that I am in an exclusive environment	
Self-esteem needs	I often have a strong need to be around people who are impressed with what I am like and what I do	Fuller et al., 2006
	I mainly like to be around others who think I am an important person	
	I mainly like to be around others who think I am an exciting person	
Recognition needs	It is important to me that other people see me as successful	Gordon and Luo, 2011
	It is important to me that other people believe in me	
	It is important to me that other people like me	
[Korean luxury cosmetic] Brand	[The Korean luxury cosmetic brand] is important to me	Brakus et al., 2009
involvement	[The Korean luxury cosmetic brand] is relevant to me	
	[The Korean luxury cosmetic brand] means a lot to me	
	[The Korean luxury cosmetic brand] is significant to me	
Informational herd behavior	To ensure that I buy the right brand, I often observe what others are buying and using	Chen, 2008
	If I have little experience with a brand, I often ask my friends about the brand	
	I frequently gather information from friends or family about a brand before I buy	
Normative herd behavior	When buying products, I generally purchase those brands that I think others will approve of	Chen, 2008
	I achieve a sense of belonging by purchasing the same brands that others purchase	
	I like to know what brands and products make good impressions on others	
Fear of missing out	I become worried when I find out my friends are having fun without me	Przybylski et al., 2013
	I become anxious when I do not know what brands are popular among my friends	
	It is important that I continue to keep in touch with what my friends are doing	

### Data Analysis Method

A SEM analysis was used for the statistical analysis, conducted through SPSS and AMOS. SEM allows us to analyze the structures describing complex multiple relationships between variables. The research model presented here has seven latent and interrelated variables. In addition, we conducted a multi-group analysis between the high-FoMO and low-FoMO groups. To do so, we compared the SEM analyses and estimated a cross-group equality constraint model between the two groups, using the same constraints for each set of parameters.

## Results

### Sample Characteristics and Correlations

Our sample contains data on 72 males (25.8%) and 207 females (74.2%). The sample size of 279 is sufficient, because a sample of at least 267 is required at the 95% confidence level, and a confidence interval of six is used for the total population of the five selected cities in China (52,781,714). Regarding the age structure, 36 respondents (12.9%) are younger than 20, 121 (43.4%) are aged 20 to 29, 108 (38.7%) are aged 30 to 39, and 14 (5.0%) are 40 or older. This is an adequate representation of the population of Korean luxury cosmetics consumers in China, because the majority are young female adults (KOTRA Report, 2019). With regard to occupation, the sample includes government employees (76, 27.2%), private firm employees (59, 21.1%), students (57, 20.4%), and the self-employed (50, 17.9%). This is also an accurate representation of the Chinese consumer population, because most people are employed by the government and private enterprises (Statista, 2019b). With regard to location, quota sampling was conducted in the five surveyed cities: 97 (35%) were sampled from Beijing, the capital; 62 (22%) were from Shanghai; 58 (20%) were from Tianjin; 43 (15%) were from Dalian; and 19 (8%) were from Shenyang (see Table 4).

**TABLE 4:** Demographic characteristics.

Item	Characteristics	Frequency	Percentage
Gender	Male	72	25.8
	Female	207	74.2
	Total	279	100
Age	19 and under	36	12.9
	20–29	121	43.4
	30–39	108	38.7
	40 and above	14	5.0
	Total	279	100
Occupation	Students	57	20.4
	Government employee	76	27.2
	Self-employed	50	17.9
	Private firm employee	59	21.1
	Others	37	13.3
	Total	279	100
Locations	Beijing	97	35
	Shanghai	62	22
	Tianjin	58	20
	Dalian	43	15
	Shenyang	19	8
	Total	279	100

Table 5 shows the means, standard deviations, and correlation matrix of the constructs used in this study. The mean of informational herd behavior (3.912) is the highest among the constructs, whereas the mean of hedonic needs is the lowest (2.853). In terms of the correlations between constructs, consumers’ hedonic needs showed the highest correlation with Korean luxury cosmetic brand involvement (0.165, *p* < 0.01). With regard to the relationship between Chinese consumers’ social identification needs and their Korean luxury cosmetic brand involvement, their need for self-esteem and for recognition were found to be the most correlated with involvement (0.305, *p* < 0.01; 441, *p* < 0.01, respectively). However, consumers’ Korean luxury cosmetic brand involvement showed a higher relevance (0.189, *p* < 0.01) for normative herd behavior than it did for informational herd behavior (0.145, *p* < 0.01).

**TABLE 5:** Correlations between constructs.

	1	2	3	4	5	6	7
1	1						
2	0.018	1					
3	0.043	–0.008	1				
4	0.144**	0.019	0.178***	1			
5	0.165***	0.111*	0.305***	0.441***	1		
6	0.069	0.034	0.187***	0.097	0.145**	1	
7	0.088	0.075	0.224***	0.227***	0.189***	0.182***	1
Mean	2.853	2.994	3.634	3.783	3.901	3.912	3.754
SD	0.937	0.981	0.821	0.785	0.756	0.979	0.828

1: hedonic needs, 2: symbolic needs, 3: recognition needs, 4: self-esteem needs, 5: (Korean luxury cosmetic) brand involvement, 6: informational herd behavior, 7: normative herd behavior. ***p < 0.01, **p < 0.05, *p < 0.1.

### Measure Validation

To test the validity of each construct, we conducted a confirmatory factor analysis (CFA) using SPSS 18.0 (see Table 6). The convergent validity was evaluated using AMOS data analysis by examining the loading of each item on its corresponding construct, thus assessing the reliability of the items. We use the following criteria for the evaluations: the composite reliability (CR), which should be at least 0.70 (Hair et al., 2011); the average variance extracted (AVE), which should exceed 0.50 (Hair et al., 2011); and all item loadings, which should be more than 0.70 (Hair et al., 2011). The CR ranges from 0.889 to 0.953, and the AVE values exceed 0.50, ranging from 0.728 to 0.872. This demonstrates the convergent validity of the measures used in this study. In addition, Hair et al. (2011) suggest that loadings over 0.30 meet the minimal level, those over 0.40 are more important, and those over 0.50 are practically significant. The loading of an item on its corresponding construct should be at least 0.32, where loadings over 0.71 are excellent, those over 0.63 are very good, those over 0.55 are good, and those over 0.45 are fair. Based on this classification, our loadings appear to be excellent. Furthermore, in terms of the constructs of the measurement items, the *t*-value of each construct is higher than 14.408, and all are statistically significant at the 99% confidence level.

**TABLE 6:** CFA results.

Constructs	Items	Factor loading	SE^a^	Std. loading	*t*-value	CR^b^	AVE^c^
Hedonic needs	HED1	1.000	–	0.781	–	0.900	0.749
	HED2	0.959	0.058	0.707	16.554		
	HED3	1.010	0.063	0.671	16.111		
Symbolic needs	SYM1	1.000	–	0.840		0.889	0.728
	SYM 2	0.922	0.054	0.805	17.102		
	SYM 3	0.910	0.053	0.806	17.103		
Self-esteem needs	SEL1	1.000	–	0.815	–	0.933	0.824
	SEL2	0.936	0.057	0.812	16.341		
	SEL3	0.859	0.053	0.805	17.786		
Recognition needs	REC1	1.000	–	0.689	–	0.907	0.765
	REC2	1.161	0.081	0.745	14.408		
	REC3	1.228	0.083	0.779	14.859		
(Korean luxury cosmetic) brand involvement	INV1	1.000	–	0.734	–	0.949	0.822
	INV2	1.114	0.062	0.721	18.017		
	INV3	1.051	0.051	0.815	20.565		
	INV4	1.059	0.050	0.738	20.998		
Informational herd behavior	IHB1	1.000	–	0.878	–	0.953	0.872
	IHB 2	0.991	0.039	0.874	25.282		
	IHB 3	1.116	0.037	0.918	30.040		
Normative herd behavior	NHB1	1.000	–	0.806	–	0.919	0.790
	NHB2	0.973	0.057	0.790	17.067		
	NHB3	1.063	0.061	0.781	17.571		

**p* < 0.01 for all loading; ^*a*^Standard error, ^*b*^Composite reliability, ^*c*^Average variance extracted.*

### Model Validation

This study uses an SEM, performed using AMOS, to examine the causal relationship between the concept variables. In general, the suitability of a model can be confirmed when all fitted indices meet the criteria shown in Table 7.

**TABLE 7:** Statistical summary of model fit.

Model goodness of fit indices	Recommended value	Results in this study
Chi-squared/degree of freedom	≤3	1.28
Goodness of Fit Index (GFI)	≥0.90	0.925
Adjusted Goodness of Fit Index (AGFI)	≥0.80	0.903
Normalized Fit Index (NFI)	≥0.90	0.942
Comparative Fit Index (CFI)	≥0.90	0.987
Incremental Fit Index (IFI)	≥0.90	0.957
Root Mean Squared Error of Approximation (RMSEA)	0.05–0.08	0.050

### Hypotheses Test Results

**FIGURE 2:**
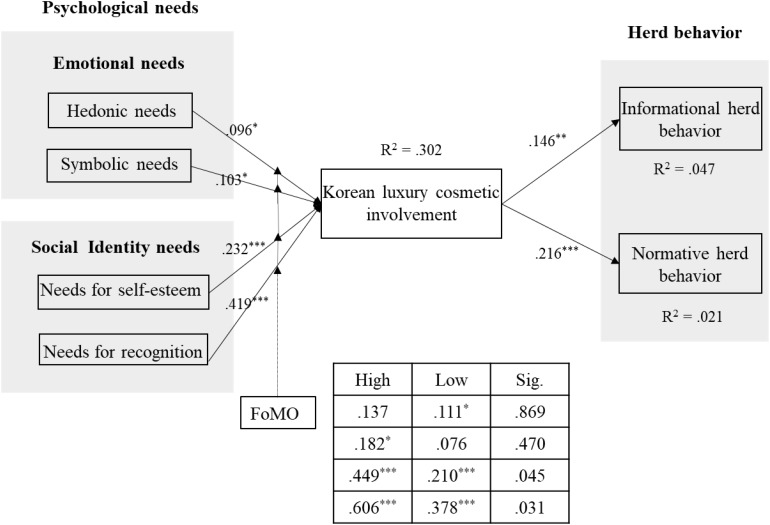
Relationships between constructs. ^∗∗∗^*p* < 0.01, ^∗∗^*p* < 0.05, ^∗^*p* < 0.1.

As shown in Figure 2, among the factors that affect brand involvement, social identification needs were found to have a higher coefficient than consumers’ emotional needs. Consumers’ symbolic needs have a positive relationship with brand involvement (0.097, *p* < 0.01). On the other hand, consumers’ social identification needs (i.e., self-esteem needs and recognition needs) were both found to have a significant and positive influence on Korean luxury cosmetic brand involvement (0.426, *p* < 0.01; 0.232, *p* < 0.01). With regard to the relationship between brand involvement and consumer herd behavior, consumers’ involvement levels were found to have a positive influence on both informational herd behavior (0.146, *p* < 0.05) and normative herd behavior (0.216, *p* < 0.01). The R^2^ value for brand involvement is 30.2%, and the informative and normative herd behavior rates are 4.7 and 2.1%, respectively. The model and path coefficients are shown in Figure 2, and the hypothesis test results are summarized in Table 8.

**TABLE 8:** Hypotheses test results.

	Hypothesis	Coefficient	Result
H1	Hedonic needs – Brand involvement	0.096*	Supported
H2	Symbolic needs – Brand involvement	0.103*	Supported
H3	Self-esteem needs – Brand involvement	0.232***	Supported
H4	Recognition needs – Brand involvement	0.419***	Supported
H5	Brand involvement – Informational herd behavior	0.146**	Supported
H6	Brand involvement – Normative herd behavior	0.216***	Supported

***p < 0.01, **p < 0.05, *p < 0.1.

### The Mediating Effect of FoMO

We conducted a multiple group analysis to investigate the influence of FoMO on the relationship between consumers’ psychological needs and Korean luxury cosmetic brand involvement. Here, we referred to the research of Przybylski et al. (2013) to divide the perceptions of FoMO into a high-level group (*N* = 97) and a low-level group (*N* = 182) and used the mean value dichotomizing method of Irwin and McClelland (2003). To carry out the multi-group analysis, we divide the SEM into two groups and estimate the cross-group equality constraint model between the two groups, with the same constraints for each set of parameters. Then, we analyze the models in each path that do not have group equivalence constraints and compare them to the chi-square values of each model to derive the difference between each path. Table 9 shows the results of the multi-group analysis in relation to the different levels of FoMO.

**TABLE 9:** Multi-group results according to FoMO.

Equality Constraint Model: *x*^2^ = 531.634 (df = 394), *x*^2^_crit_ = 1.28 (Δdf = 1, *p* < 0.01)					

Path	*x*^2^ of Equality	Δ*x*^2^	Cross-group path coefficient		Result

	Non-constraint Model		High group	Low group	
Hedonic needs – Brand involvement	531.661	0.027	0.137	0.111*	Not supported
Symbolic needs – Brand involvement	532.155	0.521	0.182*	0.076	Not supported
Self-esteem needs – Brand involvement	535.647	4.013	0.449***	0.210***	Supported
Recognition needs – Brand involvement	536.273	4.612	0.606***	0.378***	Supported

***p < 0.01, *p < 0.1.

## Conclusion and Discussion

### Theoretical and Practical Implications

This study has theoretical implications in that it investigates consumers’ motivations for herd consumption on luxury brands from the perspective of their psychological needs, including both emotional and social identity needs, by examining their brand involvement with Korean luxury cosmetic brands. Moreover, we established an empirical model in two ways, and found that social identity needs, which include self-esteem and recognition needs, have a greater influence than emotional needs do on the brand involvement level. This implies that consumers focus on what others think of them, and that their external social image is important. They seek the symbolic and image benefits of brands, which result in their involvement with certain luxury brands. Thus, recognition by others seems to have a greater effect than the need for self-esteem does.

In addition, among consumers’ emotional needs, symbolic needs showed a significant relationship with brand involvement, whereas hedonic needs did not. Because luxury brands are perceived to have a higher price and better quality than general brands, luxury brands signal consumers’ wealth and status. Consumption of luxury brands requires a certain price threshold, whereas hedonic consumption can be achieved in a variety of ways and with less expense. Furthermore, we found that consumers’ involvement with Korean luxury cosmetic brands influences two motivations for consumer herd behavior: informational herd behavior, which refers to accepting information from others as reality, and normative behavior, which refers to conforming to the expectations of others. Brand involvement was found to have a positive effect on both types of herd behavior for the luxury brands. Then, we investigated whether FoMO plays a role in the relationships between emotional satisfaction-seeking, social identification-seeking, and involvement with Korean luxury cosmetics. FoMO, which is considered relatively common in the field of psychology (Kang et al., 2019), describes people’s fear of detachment and their desire to stay continually connected with what others are doing (Przybylski et al., 2013). In contrast to many other works on FoMo as a cause of addictive online behavior, we investigate it in the context of the relationship between luxury brand evaluation and consumer involvement. Recognizing the role of FoMO in mediating consumers’ involvement levels provides a new perspective for exploring the effect of FoMO in the field of marketing.

Our results have the following managerial implications. First, we find that consumers’ emotional needs and social needs both affect their attachment to luxury brands, and that individual motivations are particularly relevant. Therefore, managers should pay attention to the psychological characteristics of consumers, and should focus particularly on the types of satisfaction and individuals’ needs for self-esteem and recognition.

Second, we proposed that Chinese consumers’ involvement with Korean luxury cosmetics has a positive influence on their herd consumption of such brands. Individuals are relatively easily influenced by others’ choices and tend to follow the common beliefs and lifestyles of the mainstream group. Thus, numerous works have examined the attitudes and ideas of other people and the choice of mainstream trends in the context of decision-making. Here, managers can use the psychology of consumers to develop strategies to promote their products and position their brands. In particular, we showed that consumers’ levels of FoMO affect the relationships between their psychological motivations and their levels of involvement. The strength of people’s fear of being excluded from a group means that individuals show a stronger tendency and willingness to change their behavior to follow that of the group. This result is supported by the characteristics of the consumers of Korean luxury cosmetics in China, who are mainly young female adults. Many consumer psychology studies report that female consumers, from their teens to their late 20s, tend to be influenced by the need to belong, which is a strong social need (Kuan et al., 2014). As a result, the effect of FoMO needs to be considered when formulating a strategy. Furthermore, advertising messages should focus on persuading those with high FoMO of the necessity of consuming the relevant products.

### Limitations and Future Research

The limitations of this study are as follows. First, this study examines Chinese consumers’ herd consumption behavior in terms of their level of FoMO and their involvement with certain brands. However, such herd consumption behavior may also be affected by the cultural and social aspects in China. Thus, future work should address the herd consumption behavior of Chinese consumers from cultural and social perspectives. Second, the mediating role of FoMO might vary depending on the characteristics of the objects under study. For example, we focused on the mediating effect of consumers’ psychological state of being eager to belong to the main group in terms of their consumption process. Young consumers might be more easily influenced by others, and the degree to which they follow the main group might be higher. Consumers’ FoMO might also affect specific characteristics, such as their age, occupation, and so on. Future works should more comprehensively examine the mediating effect of FoMO in terms of consumers’ characteristics. Third, this study selected luxury cosmetics as the research object, which is limited in terms of the categories of luxury brands. Herd consumption of luxury brands might change if different categories of brands are used. Therefore, future work should include comparative studies using different categories of products and brands. In addition, luxury cosmetics brands tend to have large discrepancies in their prices and categories. Thus, respondents’ preferred lifestyle and income levels could serve as critical control variables for future research. Fourth, our survey primarily captures a cross-sectional view of the constructs. It is possible that Chinese consumers’ perceptions of the tested luxury brands vary with time. Thus, a longitudinal study is necessary to investigate the time-variant impact of the evaluations of luxury brands. Despite these limitations, our findings are expected to add to the literature on herd consumption behavior related to luxury brands and should be useful to practitioners as well.

## Data Availability Statement

All datasets generated for this study are included in the article/supplementary material.

## Ethics Statement

The studies involving human participants were reviewed and approved by the Kyung Hee University IRB. The patients/participants provided their written informed consent to participate in this study.

## Author Contributions

All authors listed have made a substantial, direct and intellectual contribution to the work, and approved it for publication.

## Conflict of Interest

The authors declare that the research was conducted in the absence of any commercial or financial relationships that could be construed as a potential conflict of interest.
